# Exploring vaccine hesitancy among citizens of the Métis Nation of Ontario: a population-based data linkage study

**DOI:** 10.23889/ijpds.v9i5.1676

**Published:** 2024-09-18

**Authors:** Sarah Edwards, Keith King, Shelley Cripss, Ashok Chaurasia, Eliane Kim, Hira Khan, Kwong Kwong, Jennifer Walker McMaster, Martin Cooke

**Affiliations:** 1 ICES; 2 The Métis Nation of Ontario; 3 University of Toronto; 4 University of Alberta; 5 University of Waterloo

## Objective and Approach

Understanding vaccine hesitancy among distinct communities is crucial to ensuring equitable uptake of vaccination. The objective was to examine the influence of psychological antecedents of vaccine uptake, known as the “5Cs”, on COVID-19 vaccination among Métis Nation of Ontario (MNO) citizens. The Métis people are one of three Indigenous peoples in Canada. A population-based online survey was implemented by the MNO, including the short version of the “5C” (confidence, complacency, constraint, calculation, collective responsibility) psychological antecedents of vaccination scale. Census sampling achieved a 39% response rate and respondents were linked to the COVID-19 vaccine database (COVAX) in Ontario (n=4,012). Analyses included logistic regression models (adjusted for sociodemographics) and exploratory latent class analyses.

## Results

In logistic regression models, MNO citizens who were less confident that COVID-19 vaccines were safe (OR=0.04; 95% CI=0.03-0.06) and did not agree vaccination was a collective action to prevent the spread of disease (OR=0.11, 95% CI: 0.07-0.17) had lower odds of being vaccinated. MNO citizens who disagreed the risk of COVID-19 was small (OR=14.87, 95% CI: 9.99-22.13) had a higher odds of being vaccinated. Vaccine hesitancy profiles with (i) lower confidence in availability/safety of COVID-19 vaccines, (ii) lower perceptions about severity of COVID-19 and (iii) higher everyday life constraints/stressors were associated with lower vaccination rates (males, OR: 4.69; CI: 3.18–6.92; females, OR: 4.77, CI: 3.03–7.51) in latent class analyses.

## Conclusions and Implications

Métis-specific analyses enabled a community-driven response, resulting in low vaccine hesitancy and high uptake.

